# Promoting Motor Cortical Plasticity with Acute Aerobic Exercise: A Role for Cerebellar Circuits

**DOI:** 10.1155/2016/6797928

**Published:** 2016-04-04

**Authors:** Cameron S. Mang, Katlyn E. Brown, Jason L. Neva, Nicholas J. Snow, Kristin L. Campbell, Lara A. Boyd

**Affiliations:** ^1^Graduate Program in Rehabilitation Sciences, Faculty of Medicine, University of British Columbia, Vancouver, BC, Canada V6T 1Z3; ^2^Graduate Program in Neuroscience, Faculty of Medicine, University of British Columbia, Vancouver, BC, Canada V6T 1Z3

## Abstract

Acute aerobic exercise facilitated long-term potentiation-like plasticity in the human primary motor cortex (M1). Here, we investigated the effect of acute aerobic exercise on cerebellar circuits, and their potential contribution to altered M1 plasticity in healthy individuals (age: 24.8 ± 4.1 years). In Experiment*   *1, acute aerobic exercise reduced cerebellar inhibition (CBI) (*n* = 10, *p* = 0.01), elicited by dual-coil paired-pulse transcranial magnetic stimulation. In Experiment*   *2, we evaluated the facilitatory effects of aerobic exercise on responses to paired associative stimulation, delivered with a 25 ms (PAS_25_) or 21 ms (PAS_21_) interstimulus interval (*n* = 16 per group). Increased M1 excitability evoked by PAS_25_, but not PAS_21_, relies on trans-cerebellar sensory pathways. The magnitude of the aerobic exercise effect on PAS response was not significantly different between PAS protocols (interaction effect: *p* = 0.30); however, planned comparisons indicated that, relative to a period of rest, acute aerobic exercise enhanced the excitatory response to PAS_25_ (*p* = 0.02), but not PAS_21_ (*p* = 0.30). Thus, the results of these planned comparisons indirectly provide modest evidence that modulation of cerebellar circuits may contribute to exercise-induced increases in M1 plasticity. The findings have implications for developing aerobic exercise strategies to “prime” M1 plasticity for enhanced motor skill learning in applied settings.

## 1. Introduction

While aerobic exercise is commonly prescribed to promote cardiorespiratory and musculoskeletal health benefits, it is now also well-established that it exerts powerful effects on the brain [1, 2]. These aerobic exercise effects on the brain include an impact on neuroplasticity and have been largely studied in terms of chronic effects associated with long-term aerobic exercise training [1, 3, 4]. Notably, recent studies of the human sensorimotor system have utilized noninvasive brain stimulation techniques to demonstrate that a single bout of aerobic exercise can modulate plasticity in the primary motor cortex (M1) [5–7]. For example, two studies demonstrated that lower-limb cycling facilitated long-term potentiation- (LTP-) like plasticity evoked by paired associative stimulation (PAS) targeting the hand region of M1 [5, 6]. Additionally, the long-term depression- (LTD-) like effects of continuous theta burst stimulation on M1 excitability for a muscle of the hand were enhanced when stimulation was preceded by low-intensity cycling [7]. Taken together, these studies indicate that acute aerobic exercise has robust effects on neuroplasticity in humans, impacting both the up- and downregulation of M1 excitability in nonexercised muscles [5–7].

Importantly, the aforementioned work indicates that acute aerobic exercise modulates the subsequent induction of plasticity in descending M1 corticospinal projections [5–7] but that it does not itself induce plasticity in this pathway [6, 7]. For example, multiple studies determined that motor evoked potential (MEP) recruitment curve measures obtained from single-pulse transcranial magnetic stimulation (TMS) for hand muscles did not change from before to after a bout of aerobic exercise [6–9]. In contrast, current evidence suggests that intracortical excitability within hand muscle M1 representations is altered by a single bout of cycling [8, 9]. Specifically, acute aerobic exercise decreased short-interval intracortical inhibition (SICI) [8, 9] and increased intracortical facilitation (ICF) [9] for a nonexercised hand muscle, without impacting MEP recruitment curves. Taken with the evidence of exercise-induced modulation of LTP- and LTD-like M1 plasticity [5–7], these intracortical changes have been interpreted as a potential mechanism through which acute aerobic exercise may prepare the descending upper motor neurons for subsequent induction of plasticity [8, 9].

In concert with alterations in intracortical circuits [8, 9], it is plausible that changes in the excitability of neural inputs projecting from other cortical and subcortical sources to M1 could also contribute to aerobic exercise-induced increases in M1 plasticity. In a recent study examining the effects of acute aerobic exercise on motor learning, our behavioral findings suggested a possible aerobic exercise-induced potentiation of cerebellar function [6]. Specifically, high-intensity interval cycling immediately prior to practicing a continuous tracking task with a visuomotor rotation enhanced the acquisition and delayed retention of the temporal, but not spatial, element of the motor skill [6]. Cerebellar circuits are known to play an important role in motor control and learning [10], especially those involving visuomotor rotations [11, 12]. Past work in individuals with a cerebellar infarct also suggested that the learning of the temporal element of a continuous tracking task was highly dependent on cerebellar function [13]. Thus, our previous work may be taken to suggest a potential impact of acute aerobic exercise on the cerebellum [6]. Further, cerebellar circuits have been shown to modulate plasticity in M1 [14]. Nevertheless, without any direct measures of cerebellar function in our previous study [6], a postulated impact of acute aerobic exercise on cerebellar circuits is highly speculative. Therefore, the present study is comprised of two experiments designed to evaluate our hypothesis that acute aerobic exercise-induced effects on M1 may be partially mediated by cerebellar circuits projecting to M1.

The purpose of Experiment*   *1 was to examine whether activity in the cerebellothalamocortical pathway is altered by acute high-intensity aerobic exercise. We tested the activity of the cerebellothalamocortical pathway using a dual-coil paired-pulse TMS technique, termed cerebellar inhibition (CBI) [15–17], before and after both a period of rest and a bout of high-intensity aerobic exercise. We hypothesized that, similar to the decrease in SICI reported in previous work [8, 9], there would be a decrease in CBI following acute aerobic exercise. In Experiment*   *2, we examined the role of cerebellar circuits in mediating acute aerobic exercise effects on LTP-like plasticity in M1. Both studies that have previously reported a positive effect of acute aerobic exercise on responses to excitatory PAS utilized a 25 ms interstimulus interval (ISI, PAS_25_) between the peripheral and cortical stimuli [5, 6]. Interestingly, previous work demonstrated that the effects of PAS_25_ on M1 excitability are mediated in part by indirect trans-cerebellar sensory pathways [14, 18]. In contrast, it is thought that excitatory PAS with a shorter ISI (~21 ms, PAS_21_) does not provide enough time for sensory inputs via this indirect route to influence M1 and thus exerts its effects via only more direct sensory pathways (i.e., dorsal column-medial lemniscus pathway) [18]. Thus, it is possible that previously documented effects of acute aerobic exercise on PAS_25_ could be partly mediated by an aerobic exercise effect on cerebellar circuits. Here, we evaluated the impact of acute high-intensity aerobic exercise, versus a period of rest, on LTP-like plasticity in M1 evoked by both PAS_25_ and PAS_21_. Due to the involvement of cerebellar pathways, we hypothesized that the response to PAS_25_ would be facilitated by aerobic exercise to a greater extent than PAS_21_.

## 2. Methods

### 2.1. Participants

Experiments for the current study were conducted on a total of 34 young healthy participants between ages 19 and 34 (mean ± SD; 24.8 ± 4.1 years, 14 M). Participants had no known neurological disorders, were of adequate health to complete exercise protocols, and were screened for potential contraindications to TMS. All participants gave written informed consent prior to testing. The Clinical Research Ethics Board at the University of British Columbia approved all experimental procedures.

### 2.2. Experimental Design

**Figure 1:**
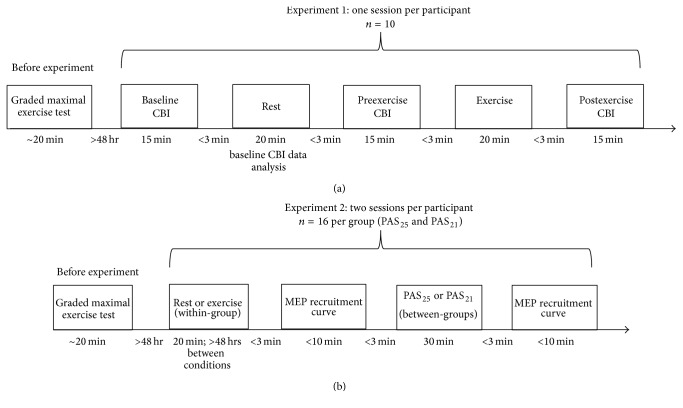
Overview of experimental procedures for Experiment*   *1 (a) and Experiment*   *2 (b). Each session was approximately two hours in duration. CBI: cerebellar inhibition; MEP: motor evoked potential; PAS_25_: paired associative stimulation with 25 ms interstimulus interval; PAS_21_: paired associative stimulation with 21 ms interstimulus interval.

This study consisted of two separate experiments designed to evaluate the potential impact of acute high-intensity cycling on M1-cerebellar circuits for a nonexercised muscle of the hand (abductor pollicis brevis, APB). Prior to participation in experimental sessions, each participant completed a graded maximal exercise test, for the purpose of subsequent exercise intensity prescription. For “Experiment*   *1,” participants completed one single session to evaluate the impact of a standardized bout of high-intensity interval cycling on CBI. The session involved an assessment of CBI at three time points: baseline, immediately following 20 minutes of seated rest (preexercise), and immediately following a 20-minute high-intensity aerobic exercise interval session (after exercise). Thirteen individuals participated in this experiment; however, the session was not completed in three participants due to a lack of CBI at the baseline time point (*n* = 2) and discomfort with cerebellar stimulation (*n* = 1). Thus, the final dataset included a total of 10 participants. For “Experiment*   *2,” 32 participants completed two sessions designed to assess the potential effects of the same high-intensity interval cycling bout on change in corticospinal excitability evoked by PAS. The experimental sessions included (1) rest followed by PAS and (2) aerobic exercise followed by PAS. Half of the participants (*n* = 16) underwent the experiments with PAS_25_ and the other half underwent PAS_21_. PAS groups were similar in terms of age, sex, cardiorespiratory fitness, and physical activity levels (Table 1). Session order was pseudo-randomized and performed at the same time of day (±2 hours) for each participant to account for diurnal fluctuations in M1 excitability [19]. Of the 32 participants involved in Experiment*   *2, 11 also participated in Experiment*   *1. On all testing days, participants were instructed to refrain from any exercise besides that involved in the experimental sessions. All sessions conducted on the same individuals were separated by at least 48 hours. The procedures are depicted in their experimental order in Figure 1.

### 2.3. Exercise Procedures

#### 2.3.1. Graded Maximal Exercise Testing

A graded maximal exercise test was conducted on a stationary cycle ergometer (Ergoselect 200, Ergoline GmbH, DE), beginning with a power output (PO) of 100 W for men and 50 W for women, and increased by 30 W increments every 2 minutes until volitional exhaustion. Participants were instructed to maintain a pedaling cadence of 70–90 revolutions per minute (RPM) and to remain seated throughout testing. During exercise testing, the following measurements were monitored: expired O_2_ and CO_2_ concentrations and air flow via a metabolic cart (ParvoMedicsTrueOne 2400, USA); heart rate (HR) via a wireless HR monitor (Polar Electro, FIN); and Borg's 6–20 scale rating of perceived exertion (RPE) [20]. Finger-stick blood lactate (BLa) was measured immediately after completion of the exercise test using an automated portable BLa analyzer and test strips (Lactate Pro, Arkray Inc., Japan). Peak O_2_ consumption (${\dot{V}O}_{2peak}$) criteria included at least one of the following: a plateau in O_2_ uptake (${\dot{V}O}_{2}$) and HR with further increase in workload, a respiratory exchange ratio greater than 1.1, a RPE greater than 17, BLa greater than 10 Mmol/L, an inability to maintain a cadence of 70 RPM, and/or volitional exhaustion. Exercise testing results averaged across the study groups are presented in Table 1.

#### 2.3.2. Standardized Acute Aerobic Exercise Bout

Maximal PO determined by the exercise test was used to inform prescription of a standardized acute aerobic exercise bout. The bout lasted 20 minutes and included a 5-minute warm-up at 50 W and self-selected cadence, followed by three 3-minute sets of high-intensity cycling interspersed with 2 minutes of low-intensity cycling. The high-intensity intervals involved cycling at 90% of maximal PO from the final fully completed stage of the maximal exercise test. The low-intensity “active rest” intervals involved cycling at 50 W. During the intervals, participants were instructed to maintain a cadence greater than 70 RPM. The aerobic exercise bout was prescribed based on previous work demonstrating systemic increases in neurochemicals with minimal long-term fatigue or dehydration [21, 22] and is similar to that previously employed by Roig and colleagues [23] and our lab [6] when examining aerobic exercise effects on motor learning and neuroplasticity.

### 2.4. Experiment*   *1

As shown in Figure 1(a), CBI was evaluated in participants at three time points over a single session: baseline, before exercise (following 20 minutes of seated rest), and after exercise. This experimental design allowed evaluation of changes in CBI following a period of seated rest (i.e., control) versus a high-intensity aerobic exercise bout.

#### 2.4.1. Cerebellar Inhibition (CBI)

Surface electromyography (EMG) was collected from 1 cm × 1 cm square surface recording electrodes (Covidien, USA) placed over the belly of the APB of the nondominant hand. EMG signals were collected using LabChart software (LabChart 7.0, AD instruments, USA) and were preamplified (1000x) and band-pass filtered at 10–1000 Hz with PowerLab amplification and EMG systems (AD instruments, USA). Data for all evoked potentials were sampled at 2000 Hz and recorded from 100 ms before to 400 ms after stimulus delivery.

TMS was first delivered using a figure-of-eight coil (Magstim 70 mm P/N 9790, Magstim Co., UK) and Magstim 200^2^ stimulator (Magstim Co., UK) over the nondominant APB M1 representation. At the baseline time point, the coil was moved over M1 to find the site that elicited the largest amplitude MEP at the lowest stimulation intensity for APB. Using Brainsight*™* image-guided neuronavigation software (Rogue Resolutions Inc., Canada) and a standardized neuroanatomical template, this stimulation site was recorded and used to maintain coil orientation for all TMS delivery. All MEPs were evoked at rest. Resting motor threshold (RMT) was determined by finding the lowest stimulation intensity that evoked MEPs of at least 50 *μ*V in 5 out of 10 consecutive trials [24].

CBI was then studied with a protocol similar to previous work [15, 25]. Cerebellar stimulation was delivered with a double cone coil (Magstim P/N 9902-00, Magstim Co., UK). The center of the double cone coil was placed 3 cm lateral (ipsilateral to the nondominant hand) of the midpoint along a line between the inion and the mastoid process. The coil junction was oriented vertically to induce an upward electric current in the underlying tissue. The conditioning stimulus (CS) was delivered through this coil immediately prior to a test stimulus (TS) delivered by the figure-eight coil placed over the nondominant APB M1 representation.

When examining CBI in previous work, the CS intensity has been commonly set to 5–10% mean stimulator output below the threshold for eliciting a cervicomedullary evoked potential (CMEP) by double cone coil stimulation over the inion [15, 25]. This past method ensures that CBI waveforms are not contaminated with CMEPs [15]. Yet CMEPs are commonly of low amplitude, can be painful for participants, and cannot be elicited in all participants [26]. Here, we opted to circumvent the CMEP threshold process and utilized RMT, determined by TMS delivery with the figure-eight coil over the APB M1 representation, as a reference point for setting the CS intensity to a sufficient intensity to elicit CBI in each participant. Of the 10 individuals that completed the experiment, a CS intensity equivalent to the previously established RMT (i.e., 1.0 × RMT) was adequate to evoke CBI in seven individuals, while a CS intensity of 1.2 × RMT was needed to evoke CBI for the other three participants. The TS was set to the intensity that evoked a MEP in the nondominant APB of 1 mV (SI 1 mV). CBI was tested at ISIs of 5, 6, and 7 ms between the CS and TS. For each ISI, a separate block of 20 stimuli was delivered involving 10 CS-TS trials flanked by five TS alone trials (i.e., 10 TS alone trials total). The order of blocks was randomized at each time point that CBI was evaluated. TS intensity was determined prior to collection of each block of stimuli. All data was collected within ~20 minutes of the completion of the rest period and aerobic exercise bout.

#### 2.4.2. Data Processing

CBI data were processed using a custom MATLAB script (MathWorks, USA). All CBI trials were inspected* post hoc* and discarded if EMG activity during the 100 ms prior to the TMS pulse for each individual trial exceeded 2 standard deviations (SD) of the average prestimulus signal. Data were also visually inspected* post hoc* and trials removed in instances that the CS elicited corticospinal, cervical root, or antidromic activity [27]. Less than 0.1% of all responses were removed from further analyses based on these criteria. CBI was determined for each ISI at each time point as the ratio of the mean conditioned MEP amplitude to the mean unconditioned MEP amplitude collected in the same block of stimuli, where a lower value indicates more CBI. Baseline CBI data were analyzed online. Initially, five participants did not demonstrate CBI at baseline with a CS intensity of 1.0 × RMT and were retested for CBI with a CS intensity of 1.2 × RMT. Three of these individuals demonstrated CBI at this higher CS intensity and were tested at the remaining experimental time points with the higher CS intensity. Two participants did not show CBI even at the higher CS intensity and thus their experimental sessions were discontinued following the baseline measurement. CBI was considered present at baseline, and the experiment continued, if a one-sample one-direction *t*-test indicated that CS-TS trials were on average lower than TS alone trials in the same block at any ISI at baseline (*p* < 0.05). The ISI that yielded the lowest CBI ratio at baseline was identified for each individual. CBI at this specific ISI for each individual was then compared between time points in the statistical analyses. Previous work has recommended that, in order to measure a change in CBI induced by an intervention (i.e., aerobic exercise), it is necessary to initially conduct CBI procedures with parameters stimulus parameters that yield an approximate 50% suppression of the TS [27]. The above described procedures ensured that CBI was evoked at approximately this amplitude in each participant at the outset of the experiment.

#### 2.4.3. Statistical Analyses

A one-way repeated measures analysis of variance (RM-ANOVA) was conducted with the factor time (baseline, before exercise, and after exercise) to ensure that TS amplitude was similar across time points. Next, to evaluate whether aerobic exercise impacted CBI, a second one-way RM-ANOVA with factor time (baseline, before exercise, and after exercise) was conducted with CBI ratio as the dependent variable.* Post hoc* Tukey's HSD tests were conducted on the main effect of time.

Following visual inspection for skewness and kurtosis and objective testing for normality with the Shapiro-Wilk test with a significance level set at *p* < 0.001 [28], all variables were found to be normally distributed (*W*_(10)_ ≥ 0.916, *p* ≥ 0.33). For all statistical tests, significance level was *p* < 0.05. All descriptive statistics are reported as mean ± SD in the text. Statistical analyses were conducted using SPSS (v. 23.0, IBM Corporation, USA) and Statistica (v. 12.0, Statsoft Inc., Dell Software, USA) software.

### 2.5. Experiment*   *2

The following procedures were conducted with each participant under each experimental condition (rest and aerobic exercise). The order of conditions was randomized for each participant (Figure 1(b)). During Experiment*   *2, EMG was collected as described above for Experiment*   *1.

#### 2.5.1. Median Nerve Stimulation

Rectangular pulses of 0.2 ms duration were delivered over the median nerve at the wrist of the nondominant hand using a constant current stimulator (DS7A, Digitimer, UK). Immediately before MEP recruitment curve collection (see below), electrical stimulation intensity was increased over 5–10 stimuli from below motor threshold to 1.5 times the minimum current to evoke the maximal M-wave (M_max_) in APB. M_max_ was determined as the largest peak-to-peak amplitude M-wave evoked in APB in these stimuli. M_max_ is a stable measure of muscle activity during maximal muscle fibre recruitment [29] and was used as a reference from which to normalize MEPs evoked by TMS [6, 7].

#### 2.5.2. Motor Evoked Potential (MEP) Recruitment Curves

The figure-of-eight coil (Magstim 70 mm P/N 9790, Magstim Co., UK) was used to locate the nondominant APB hotspot and RMT, as above. Next, a MEP recruitment curve (pre-PAS) determined corticospinal excitability via measurement of the amplitude of MEPs elicited at varying TMS intensities. Ten stimuli were delivered at 0.25 Hz in a random order at intensities ranging from 90 to 150% of RMT, in 10% increments for a total of 70 stimuli collected over ~5 minutes [30]. Recruitment curves were collected using the same stimulation site and intensities immediately before PAS (beginning within ~3 minutes following rest or exercise) and after PAS (beginning within ~3 minutes after PAS). Including the delivery of PAS, all assessments were completed within ~45 minutes following the rest period or aerobic exercise bout.

#### 2.5.3. Paired Associative Stimulation (PAS)

Electrical stimulation was delivered over the median nerve of the nondominant limb with 0.2 ms duration pulses at 300% perceptual threshold 25 ms (PAS_25_) or 21 ms (PAS_21_) prior to delivery of suprathreshold single-pulse TMS. TMS was applied over the APB M1 representation for the nondominant limb at an intensity that evoked a MEP of approximately 1 mV (SI_1mV_). In total, 450 paired stimuli were delivered at 0.25 Hz (30 minutes of stimulation). Similar PAS protocols have previously been shown to enhance corticospinal excitability [6, 31, 32].

#### 2.5.4. Data Processing

MEP recruitment curve data were processed using a custom MATLAB script (MathWorks, USA). As in Experiment*   *1, MEPs were inspected* post hoc* and discarded in the case of EMG activity prior to the TMS pulse (<0.5% of responses removed). Plots of stimulation intensity (% RMT) by MEP amplitude (peak-to-peak amplitude expressed as % M_max_) were constructed for each individual at each time point and under each condition. As with previous work [6, 31, 33], a linear regression line was fit to the MEP recruitment curve plots (90–150% RMT), with a larger recruitment curve slope value following PAS indicating an increase in corticospinal excitability.

#### 2.5.5. Statistical Analyses

To determine whether M_max_ amplitude changed across time in each experimental session, paired samples *t*-tests were conducted for each condition and each PAS group on pre-PAS and post-PAS time points. PAS parameters were also tested for any potential differences between conditions (rest and aerobic exercise) and PAS groups (PAS_25_ and PAS_21_). Two-way mixed ANOVAs were performed to compare the 300% PT stimulation intensity (mA), RMT (% mean stimulator output, MSO), SI_1mV_ intensity (% MSO), and pre-PAS recruitment curve slope between the conditions and PAS groups.

A two-way mixed ANOVA was conducted to evaluate the impact of aerobic exercise on PAS response. The dependent variable was percent change in recruitment curve slope from before PAS to after PAS. The within-subject factor was condition (rest and exercise) and the between-groups factor was PAS group (PAS_25_ and PAS_21_). Given evidence from Experiment*   *1 that activity in the cerebellothalamocortical pathway may be modulated by exercise and our hypothesis that acute aerobic exercise would facilitate response to PAS_25_ to a greater extent than PAS_21_, we conducted planned comparisons to evaluate the difference in change in recruitment curve slope evoked under the rest and aerobic exercise conditions for each PAS group separately. To further explore any potential differences in the magnitude of the effect of aerobic exercise on response to PAS_25_ versus PAS_21_, effect sizes (*η*_partial_^2^) were calculated on the difference in PAS-induced change in MEP recruitment curve slope between the rest and aerobic exercise conditions separately for the PAS_25_ and PAS_21_ groups. Effect size calculations were interpreted based on previously developed guidelines [34].

Using criteria as described above in Experiment*   *1, all variables were found to be normally distributed (*W*_(16)_ ≥ 0.894, *p* ≥ 0.06). All statistical tests were conducted with a significance level of *p* < 0.05, all descriptive statistics are reported as mean ± SD, and statistical analyses were conducted using SPSS (v. 23.0, IBM Corporation, USA) and Statistica (v 12.0, Statsoft Inc., Dell Software, USA) software.

## 3. Results

### 3.1. Experiment*   *1

**Figure 2:**
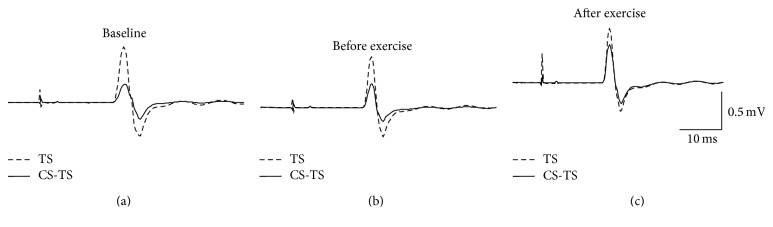
CBI in a single participant at (a) baseline, (b) before exercise, and (c) after exercise. MEP waveforms are averaged from 10 MEPs. Dashed waveforms show MEPs when the TS is delivered alone. Solid line waveforms show MEP waveforms when the TS was preceded by a CS delivered over the cerebellum. For this individual, the CS was delivered at RMT and preceded the TS by 7 ms. TS: test stimulus; CS: conditioning stimulus.

**Figure 3:**
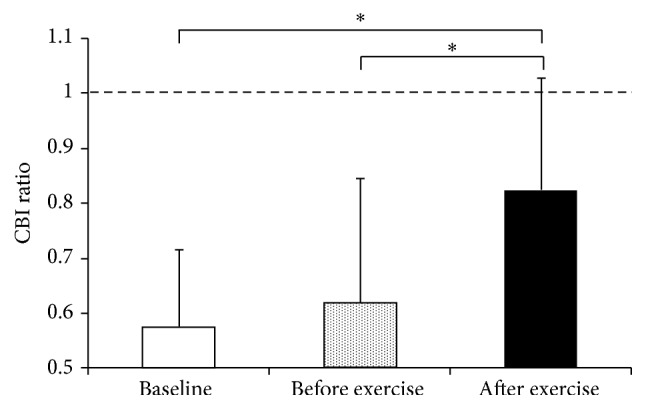
CBI ratios averaged across the group. A value of 1.0 on the *y*-axis (depicted by dashed line) indicates the amplitude of the TS alone. Asterisks indicate statistical significance (*p* < 0.05). Error bars represent one standard deviation. CBI: cerebellar inhibition.

TS amplitude during CBI collection did not change across experimental time points (*F*_(2,18)_ = 1.07, *p* = 0.37, baseline: 0.99 ± 0.51 mV, before: 1.22 ± 0.50 mV, and after: 1.12 ± 0.55 mV).  Figure 2 shows mean MEP waveforms collected for CBI in a single participant at each time point.  Figure 3 depicts the CBI ratios averaged across the group at each time point. The one-way RM-ANOVA conducted on CBI ratio detected a significant main effect of time (*F*_(2,18)_ = 6.11, *p* = 0.01).* Post hoc* analyses indicated that CBI ratio was significantly higher following aerobic exercise compared to the baseline (*p* = 0.01) and preexercise time points (*p* = 0.04). In contrast, CBI ratio did not change from before to after the period of seated rest (baseline to before exercise, *p* = 0.84).

### 3.2. Experiment*   *2

M_max_ did not change across time (before PAS and after PAS) in the rest or aerobic exercise conditions in either the PAS_25_ or PAS_21_ groups (*t*_(15)_ ≤ |1.62|, *p* ≥ 0.13). Additionally, there were no effects of condition, PAS group, or interactions for 300% PT, RMT, SI_1mV_ intensities, and pre-PAS MEP recruitment curve slope (*F*_(1,30)_ ≤ 1.42, *p* ≥ 0.24). These analyses indicate that when considering the entire study sample, there were no differences in PAS procedures or initial MEP recruitment curve slope across conditions (rest and aerobic exercise) or PAS group (PAS_25_ and PAS_21_).

**Figure 4:**
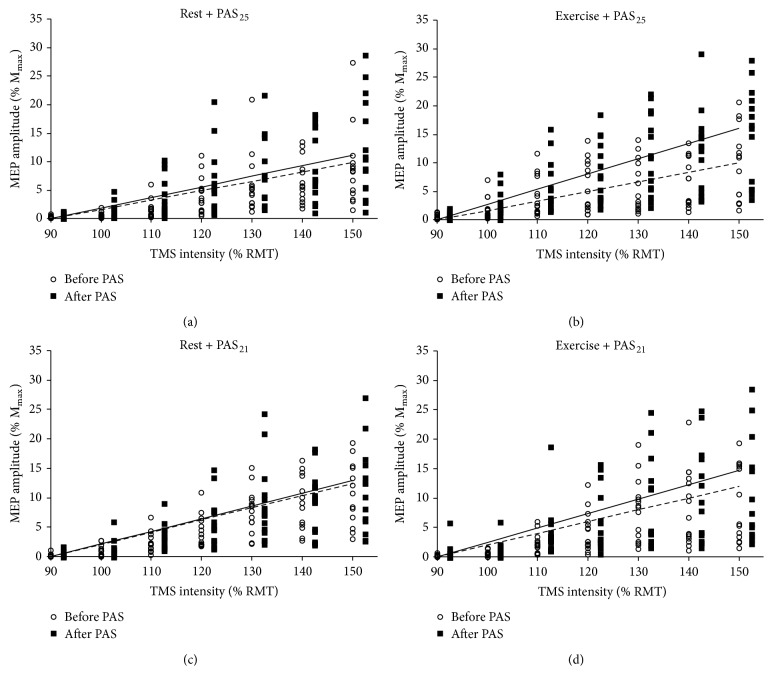
MEP recruitment curve data before and after PAS under rest (a and c) and aerobic exercise conditions (b and d) for each participant in both the PAS_25_ (a and b) and PAS_21_ (c and d) groups. Unfilled circles and filled squares depict MEP amplitude at each stimulator intensity for each participant at pre- and post-PAS time points, respectively. Likewise, dashed and solid lines depict linear regression lines showing average recruitment curve slope for the group at pre- and post-PAS time points, respectively. MEP: motor evoked potential; M_max_: maximal motor-wave; PAS_25_: paired associative stimulation with 25 ms interstimulus interval; PAS_21_: paired associative stimulation with 21 ms interstimulus interval; TMS: transcranial magnetic stimulation; RMT: resting motor threshold.

**Figure 5:**
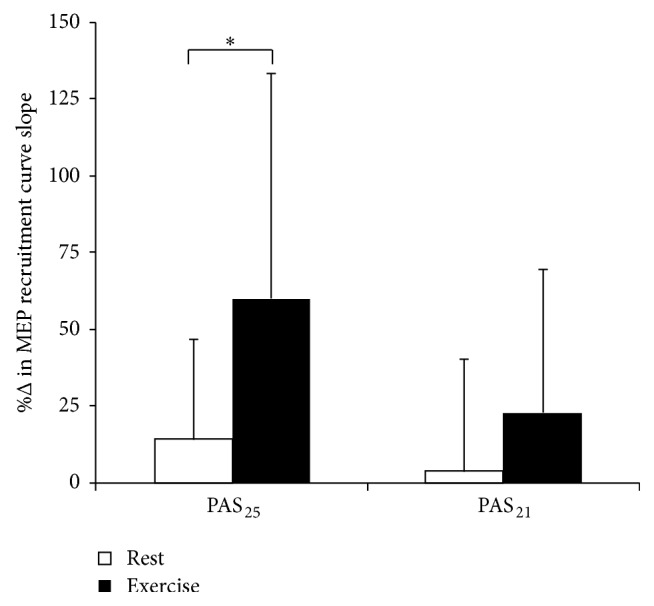
Change in MEP recruitment curve slope evoked by PAS under rest and aerobic exercise conditions. Asterisks indicate statistical significance (*p* < 0.05). Error bars represent one standard deviation. MEP: motor evoked potential; PAS_25_: paired associative stimulation with 25 ms interstimulus interval; PAS_21_: paired associative stimulation with 21 ms interstimulus interval.

Figure 4 shows the data points comprising pre-PAS and post-PAS MEP recruitment curve plots for each participant from both the PAS_25_ and PAS_21_ groups under each condition. The group average linear regression lines for the MEP recruitment curve plots, depicting the slope across the study sample at pre-PAS and post-PAS time points under each condition and in each PAS group, are also depicted in Figure 4. The mixed ANOVA detected a significant main effect of condition on change in recruitment curve slope evoked by PAS (*F*_(1,30)_ = 6.49, *p* = 0.02), a trend for an effect of PAS group (*F*_(1,30)_ = 3.75, *p* = 0.06), and no interaction effect (*F*_(1,30)_ = 1.10, *p* = 0.30). The hypothesis that the magnitude of the acute aerobic exercise effect on PAS response would differ between PAS_25_ and PAS_21_ protocols was tested by planned comparisons. MEP recruitment curve slope was increased to a greater extent by PAS_25_ under the aerobic exercise condition (59.8  ±  73.5% increase) compared to the rest condition (14.2 ± 32.7% increase; *F*_(1,30)_ = 6.47, *p* = 0.02), but there was no significant difference between conditions in the magnitude of change evoked by PAS_21_ (rest: 3.7 ± 36.3%, aerobic exercise: 22.7 ± 46.7%; *F*_(1,30)_ = 1.12, *p* = 0.30) (Figure 5). Further, effect size calculations indicated that aerobic exercise had a large facilitatory effect on response to PAS_25_ (*η*_partial_^2^ = 0.27) and a small-moderate facilitatory effect on response to PAS_21_ (*η*_partial_^2^ = 0.09).

## 4. Discussion

We conducted two experiments to examine (1) the impact of acute aerobic exercise on the excitability of cerebellar circuits and (2) the potential role of cerebellar circuits in mediating acute aerobic exercise-induced modulation of M1 plasticity. In Experiment*   *1, we found that CBI was decreased immediately following acute high-intensity aerobic exercise. In Experiment*   *2, planned comparisons revealed that the M1 excitatory response to PAS_25_, but not PAS_21_, was significantly facilitated by acute aerobic exercise; however, the magnitude of the exercise effect on PAS response was not significantly different between PAS protocols (i.e., nonsignificant interaction). Previous work demonstrated that the LTP-like effects of PAS_25_, but not PAS with shorter ISIs (i.e., PAS_21_), on M1 excitability are partly mediated by cerebellar circuits [18]. Thus, the present work suggests that acute aerobic exercise modulates activity in the cerebellothalamocortical circuit (Experiment*   *1) and provides modest evidence that cerebellar circuits may contribute to aerobic exercise-induced facilitation of LTP-like plasticity in M1 (Experiment*   *2). These findings have implications for understanding the motor circuits underpinning acute aerobic exercise influences on neuroplasticity in M1.

### 4.1. Experiment*   *1

The majority of research investigating the mechanisms by which acute aerobic exercise affects the brain has focused on the role of neurochemicals, such as catecholamines and neurotrophic growth factors, which are transiently elevated after a bout of aerobic exercise [22, 35–38]. These increases in neurochemicals are thought to promote the development of a cortical environment that is supportive of plasticity and, hence, receptive to meaningful experience (e.g., cognitive training, skilled motor practice) [39, 40]. The contention that acute aerobic exercise creates a particularly neuroplastic milieu in the cortex is further supported by work demonstrating reduced SICI [8, 9] and enhanced ICF [9] in M1 representations for nonexercised muscles. SICI and ICF reflect activity of GABA (*γ*-amino butyric acid) [41] and NMDA (*N*-methyl_D_-aspartate) [42] receptors, both of which are highly implicated in the induction of LTP-like plasticity in M1 [43, 44]. Thus, while acute aerobic exercise alone does not necessarily induce neuroplasticity in M1, it appears to prepare or “prime” the brain for plasticity to occur. This notion is consistent with the principle of “gating” of plasticity, which proposes that the induction of synaptic plasticity is dependent on the excitability of the stimulated neurons [45]. In the current paradigm, a weakening of M1 intracortical inhibitory and strengthening of facilitatory circuits by acute aerobic exercise [8, 9] concurrent with delivery of a plasticity induction protocol to M1 (PAS) would then be expected to “gate” (i.e., facilitate) the PAS effects.

The results from Experiment*   *1 demonstrate that acute aerobic exercise may also modulate activity in cerebellar circuits that project to M1. Specifically, we found that CBI in a nonexercised muscle of the hand was reduced immediately following a single bout of high-intensity cycling (Figure 3). CBI, as measured with dual-coil paired-pulse TMS, involves cerebellar stimulation 5–7 ms prior to M1 stimulation, resulting in a suppression of the MEP elicited by M1 stimulation alone [15–17, 46]. The cerebellar stimulation is thought to activate Purkinje cells, which inhibit the tonic excitatory drive from the dentate nucleus to M1 via the ventral lateral thalamus [10, 15, 16]. Previous studies have confirmed this CBI circuit, ruling out a potential effect of the cerebellar stimulus on activation of the brachial plexus, corticospinal tract, or other potential subcortical influences [16, 47]. Past work has also indicated no direct effect of acute aerobic exercise on corticospinal excitability [6–9] or spinal excitability [48] of nonexercised upper limb muscles, suggesting that the observed release of CBI was likely not mediated by changes at these sites. However, interactions between interneuronal populations involved in SICI and those which receive cerebellar projections must also be considered [17]. Specifically, a contribution of reduced SICI after acute aerobic exercise [8, 9] in mediating the presently observed reduction in CBI cannot be entirely excluded. Yet SICI represents one of many interneuronal populations in M1 and is likely not the sole recipient of cerebellar inputs to M1 [17, 41]. Regardless, our results indicate that inhibition of M1 induced by activation of the cerebellothalamocortical pathways is reduced immediately following acute aerobic exercise.

The driving force behind modulation of neural inputs to M1 by acute aerobic exercise may relate back to aforementioned changes in catecholamines and neurotrophic growth factors in the brain following aerobic exercise [22, 35–38]. For example, animal work has demonstrated aerobic exercise-induced elevations in brain-derived neurotrophic factor (BDNF) in multiple brain regions, including the cerebellum [49]. Nevertheless, when considering previous reports of reduced SICI after aerobic exercise [8, 9], it seems that a more likely mechanism for the present finding of reduced CBI may relate to an impact of aerobic exercise on GABA. GABA is the chief inhibitory neurotransmitter in the central nervous system and both inhibitory interneurons in M1 [41] and Purkinje cells in the cerebellum are GABAergic [50]. A shift from inhibitory to excitatory neurotransmitters immediately after aerobic exercise could then account for both intracortical [8, 9] and the cerebellothalamocortical changes reported here. However, without a direct measure of GABA levels in the central nervous system, we cannot conclusively determine its involvement in the presently reported effects. Nevertheless, such changes in excitatory and inhibitory neurotransmitter systems could contribute towards the creation of a favorable M1 environment for induction of plasticity, as suggested in other works [5–7].

### 4.2. Experiment*   *2

In Experiment*   *2, we examined the effects of acute aerobic exercise on changes in corticospinal excitability evoked by PAS_25_ and PAS_21_. Although there is some evidence for involvement of spinal circuits [51], changes in corticospinal excitability evoked by PAS are thought to largely reflect alterations in M1 excitability, given a lack of change in F-waves and potentials evoked by electrical brainstem stimulation following PAS [32]. PAS exerts its effects on M1 excitability via spike-timing-dependent plasticity (STDP), with excitatory effects evoked when the ISI between the sensory and cortical stimuli is equal to or slightly greater than the latency of the N20 sensory evoked potential (i.e., the time for a sensory volley to reach M1) [52]. As such, excitatory PAS is typically delivered using ISIs ranging from approximately 21 ms to 25 ms, with the sensory input generally thought to reach M1 by rapid conduction via the dorsal column-medial lemniscal system to the sensory thalamus, followed by either direct thalamic connections or via somatosensory cortex [18, 32, 52–55]. Importantly though, past work demonstrated that both anodal and cathodal transcranial direct current stimulation of the cerebellum blocks PAS_25_ effects on M1 excitability but has no effect on response to excitatory PAS delivered with a slightly shorter ISI of 21.5 ms [18]. Further work has demonstrated that continuous theta burst stimulation of the cerebellum modulates changes in M1 excitability evoked by PAS_25_ [14]. Thus, PAS_25_ appears to utilize trans-cerebellar sensory pathways to enhance M1 excitability, in addition to the more direct sensory pathways involved in PAS with slightly shorter ISIs [18]. This interpretation is consistent with animal work demonstrating indirect sensory pathways to M1 that travel through the cerebellum [56–58].

Although a test of the interaction effect in the mixed ANOVA was nonsignificant and suggests that the magnitude of the acute aerobic exercise effect on PAS response was not significantly different between PAS protocols, planned comparisons demonstrated that acute aerobic exercise significantly facilitated response to PAS_25_, but not PAS_21_ (Figure 5). Also, effect size calculations showed that the aerobic exercise bout had a large effect on response to PAS_25_ (*η*_partial_^2^ = 0.27) and a small-moderate effect on the PAS_21_ protocol (*η*_partial_^2^ = 0.09). Thus, the results of these planned comparisons indirectly suggest that the facilitatory effect of acute aerobic exercise on PAS response was at least partly dependent on the PAS protocol that was employed. As the primary mechanistic difference between PAS_25_ and PAS_21_ is the involvement of a trans-cerebellar sensory pathway [18], this finding combined with an acute aerobic exercise effect on CBI in Experiment*   *1 suggests that activity in the cerebellothalamocortical pathway is modulated by acute aerobic exercise and may subsequently play a role in facilitating M1 plasticity. Nevertheless, it is unlikely that the effects of acute aerobic exercise on response to excitatory PAS of M1 are mediated solely by excitability changes in cerebellar circuits. Although acute aerobic exercise did not significantly facilitate PAS_21_ response, effect size calculations of the exercise influence on the PAS_21_ protocol alone demonstrated that aerobic exercise still had a facilitatory effect of nearly moderate magnitude [34]. Thus, it is more probable that excitability changes in this trans-cerebellar sensory pathway summated with changes in other motor circuits, such as SICI and ICF [8, 9], to amplify the facilitatory effects of aerobic exercise on M1 plasticity evoked by PAS_25_, compared to PAS_21_.

We also observed a trend (*p* = 0.06) for a main effect of PAS protocol, indicating that the change in slope evoked by PAS was greater with PAS_25_ compared to PAS_21_ when collapsed across conditions (rest and aerobic exercise). Participant characteristics and experimental procedures were comparable between PAS groups and are unlikely to have contributed to this trend. Importantly, the trend for an effect of PAS protocol appears to be driven by the difference between PAS groups under the aerobic exercise condition (PAS_25_: 59.8 ± 73.5%, PAS_21_: 22.7 ± 46.7% increases), rather than the rest condition (PAS_25_: 14.2 ± 32.7%, PAS_21_: 3.7 ± 36.3% increases). However, a slight difference in the magnitude of the PAS effect at rest may relate to differences in STDP between PAS_25_ and PAS_21_. For example, PAS_21_ may be on the cusp of the appropriate ISI range to produce LTP-like effects and consequently elicit smaller effects. Further, the involvement of additional sensory pathways in PAS_25_ compared to PAS_21_ [18] could also plausibly affect the size of the resting PAS response. Regardless, we were not interested in comparing the magnitude of the PAS response between protocols, but rather in comparing the magnitude of the aerobic exercise effect on PAS response between the protocols.

### 4.3. Limitations

Although participants were instructed to minimize upper limb muscle activity during the aerobic exercise bout, EMG activity was not monitored. Thus, it is possible that gripping of the cycle ergometer handle bars could have contributed to the observed effects in both experiments. Also, it is difficult to definitively determine what aspects of the aerobic exercise bout caused the observed effects; for example, whether the results might be influenced by fatiguing, rather than nonfatiguing, or active versus passive leg movements cannot be elucidated from the current experimental design. Nevertheless, given the accumulating evidence for acute aerobic exercise effects on excitability of intracortical circuits [8, 9] and neurochemicals [36], we suggest that the observed effects are more likely related to a direct impact of aerobic exercise on the brain. In Experiment*   *2, an exercise-induced change in arousal and/or attention [59] could have influenced our results, as a response to PAS has been previously shown to depend on attention to the stimuli [60]. However, participants were not instructed to attend to the PAS stimuli under either condition or in either PAS group. Also, Singh and colleagues [5] demonstrated a similar facilitatory effect of acute aerobic exercise, compared to rest, on PAS_25_ response when participants' attention levels were monitored, suggesting that attentional changes likely do not drive these acute aerobic exercise effects. Nevertheless, our decision to not instruct participants to attend to the PAS stimuli may have attenuated the PAS responses observed in our study. Finally, we tested different participants for the PAS_25_ and PAS_21_ protocols. Utilizing a full repeated measures experimental design would have removed any potential influence of participant characteristics on the magnitude of the aerobic exercise effect; however, the characteristics of the groups were well-matched (i.e., age, sex, cardiorespiratory fitness, and physical activity levels). Therefore, despite this limitation, we are confident that the current results were not significantly influenced by our experimental design.

### 4.4. Implications

The impact of acute aerobic exercise on neuroplasticity in human M1 is a relatively recent discovery [5–7] that has led to speculation that acute aerobic exercise may be used to “prime” the learning of motor skills in sport and neurorehabilitation settings [39, 40]. This idea is supported by work showing benefits of acute aerobic exercise on motor learning tasks [6, 23]. Our current findings, suggesting an impact of acute aerobic exercise on cerebellar circuits, are in line with the results of our previous behavioral experiments [6] that demonstrated an effect of high-intensity aerobic exercise specifically on complex motor task elements associated with cerebellar function [13]. Interestingly, reductions in CBI, similar to those observed in Experiment*   *1 following high-intensity aerobic exercise, have also been demonstrated following the learning of a locomotor adaptation [61] and a visual hand perturbation task [62]. Thus, acute aerobic exercise may have the capacity to initiate physiological processes in the cerebellum that underpin motor learning. Given our findings here, further studies might consider whether the learning of motor tasks known to involve cerebellar function is particularly amenable to augmentation by acute aerobic exercise. It may also be of interest to consider how acute aerobic exercise impacts the sensorimotor system specifically in individuals with cerebellar damage. For example, cerebellar stroke influences the manner in which complex motor skills are learned [13]; perhaps, aerobic exercise has potential to normalize such deficits.

### 4.5. Conclusions

This study suggests that acute aerobic exercise may impact the excitability of cerebellar circuits (Experiment*   *1) and provides modest evidence that cerebellar circuits may contribute to exercise-induced increases in LTP-like plasticity in M1 (Experiment*   *2). Taken with previous work investigating intracortical M1 excitability after acute aerobic exercise [8, 9], our results suggest that aerobic exercise may promote a somewhat global decrease in inhibitory input to M1, which could contribute to the creation of a favorable neural environment for the induction of LTP-like plasticity.

## Figures and Tables

**Table 1:** Participant characteristics.

	Experimental group		
	CBI	PAS_25_	PAS_21_
*n*	10	16	16
Age	24.5 ± 3.3	23.9 ± 3.7	25.7 ± 4.6
Sex	7 F	8 F	10 F
Exercise test, final stage			
${\dot{V}O}_{2peak}$	45.3 ± 11.4	45.3 ± 9.5	43.3 ± 8.4
PO	227 ± 62.9	221 ± 66.1	220 ± 61.7
HR	181 ± 10.4	185 ± 6.4	181 ± 10.6
RER	1.20 ± 0.16	1.19 ± 0.08	1.17 ± 0.06
BLa	11.6 ± 2.0	12.4 ± 2.1	12.8 ± 2.2
RPE	18.4 ± 1.5	18.3 ± 1.6	18.2 ± 1.3
Exercise bout			
PO	205 ± 61.4	197 ± 61.8	195 ± 58.9
HR	170 ± 7.9	178 ± 11.7	170 ± 10.7
BLa	11.9 ± 4.1	11.9 ± 3.2	13.6 ± 3.8
RPE	15.3 ± 2.2	15.2 ± 1.6	15.1 ± 1.7

${\dot{V}O}_{2peak}$: peak oxygen consumption (mL/kg/min); PO: power output (W); HR: heart rate (beats/min); RER: respiratory exchange ratio; BLa: blood lactate (Mmol/L); RPE: Borg's rating of perceived exertion (6–20-point scale). PO in the “exercise bout” section was consistent across all three high-intensity intervals for each bout; the remaining values were collected at the end of the third (final) interval within an exercise bout.
